# Chromosomal Location of Genes Differentially Expressed in Tumor Cells Surviving High-Dose X-ray Irradiation: A Preliminary Study on Radio-Fragile Sites

**DOI:** 10.3390/cimb43020080

**Published:** 2021-09-08

**Authors:** Kaori Tsutsumi, Moe Masuda, Hiroyuki Date

**Affiliations:** 1 Faculty of Health Sciences, Hokkaido University, Sapporo 060-0812, Japan; date@hs.hokudai.ac.jp; 2 Tomakomai City Hospital, Tomakomai 053-8567, Japan; m.masuda@tomakomai-city-hospital.com

**Keywords:** X-ray irradiation, tumor cells, fragile site, chromosome

## Abstract

Altered gene expression is a common feature of tumor cells after irradiation. Our previous study showed that this phenomenon is not only an acute response to cytotoxic stress, instead, it was persistently detected in tumor cells that survived 10 Gy irradiation (IR cells). The current understanding is that DNA double-strand breaks (DSBs) are recognized by the phosphorylation of histone H2AX (H2AX) and triggers the ataxia-telangiectasia mutated (ATM) protein or the ATM- and Rad3-related (ATR) pathway, which activate or inactivate the DNA repair or apoptotic or senescence related molecules and causes the expression of genes in many instances. However, because changes in gene expression persist after passaging in IR cells, it may be due to the different pathways from these transient intracellular signaling pathways caused by DSBs. We performed microarray analysis of 30,000 genes in radiation-surviving cells (H1299-IR and MCF7-IR) and found an interesting relation between altered genes and their chromosomal loci. These loci formed a cluster on the chromosome, especially on 1q21 and 6p21-p22 in both irradiated cell lines. These chromosome sites might be regarded as “radio-fragile” sites.

## 1. Introduction

X-rays are widely used in various fields, especially in medicine for diagnostic and therapeutic purposes. There have been tremendous advances in these technologies, and the applications of radiation in medicine continue to increase. At the same time, there has been increased focus on the importance of understanding the effects of radiation on the human body, such as the mechanisms underlying radiation-induced DNA double-strand breaks (DSBs), radiation-induced signal transduction, and/or alterations in gene expression, which have been well studied [1,2,3]. On the other hand, the emergence of tolerant tumor cells during or after radiotherapy remains problematic, and the characteristics of these tumor cells remain unclear.

Genomic and chromosomal instability resulting from various mutational processes occurring within the genome and chromosomes leads to alteration in the expression of tumor-suppressor genes and is associated with tumor progression and tumor malignancy [4]. Radiation also triggers genomic changes as well as changes in gene expression and cellular characteristics [1,5,6,7,8]. Muradyan et al. reported that high-dose X-rays induced genomic changes [9]. Some forms of genomic and chromosomal instability result in common genomic or chromosomal fragile sites [9]. Sarni et al. reported that there are common fragile sites that are susceptible to replication stress and are hotspots for chromosomal instability in cancer [10].

On the other hand, the number of radiation-induced DNA DSBs correlates well with probabilistic models of energy transfer to DNA by secondary electrons derived from ionizing radiation [1,11,12]. Therefore, we hypothesized the possibility of the presence of the common fragile chromosomal sites that are easy targets for radiation, and these may lead the radio-tolerant tumor cells during or after radiotherapy. In our previous study, we established a subclone of the p53-null non-small cell lung cancer cell line H1299 (H1299-IR) that survived 6 MV of 10 Gy X-rays and its increased cellular motility, invasiveness, and adhesion and the change of the gene expression were reported [7,8]. However, the mechanism how IR cells obtain their changes of the characteristics, and the gene expression has been unknown. There are several possible reasons for them: for example, IR cells may possess the genotype that can survive X-ray irradiation; they may be cancer stem cells; the accumulation of the DNA repair error may have occurred after DSB or SSB induction; and so on. As for the changes of the gene expression in IR cells, it is interpreted that different pathways from the DSBs based signal transduction may have arisen because the changes persist after passaging the cells. Among the abundant information on the changes of the gene expression obtained by microarray analysis, the present study focused on the relation between changes of the gene expression and their chromosomal locations.

In the present study, we preliminarily searched for fragile sites on tumor cells that survived 10 Gy X-ray irradiation by gene locus mapping via microarray analysis using H1299-IR and MCF7-IR cells.

## 2. Materials and Methods

### 2.1. Cell Culture

The human non-small cell lung cancer cell line H1299 and the human breast cancer cell line MCF7 were obtained from American Type Culture Collection (ATCC, Manassas, VA, USA) and Health Science Research Resources Bank (JCRB, Osaka, Japan), respectively. Both lines were maintained in Dulbecco’s modified Eagle’s medium (DMEM, Sigma, St. Louis, MO, USA) supplemented with 10% fetal bovine serum (FBS; Nichirei Biosciences Inc., Tokyo, Japan) at 37 °C in a humidified atmosphere of 95% air and 5% CO_2_. To establish radiation-surviving cells, semi-confluent cell cultures were exposed to 10 Gy X-rays at room temperature using a linear accelerator (Mitsubishi Electric Co., Tokyo, Japan). The cells were immediately dispersed with 0.25% trypsin-EDTA (Sigma, St. Louis, MO, USA) and the cells were roughly spread onto culture dishes (100-mm diameter). After 14–30 days, all colonies were harvested and referred to as IR cells (i.e., H1299-IR and MCF7-IR).

### 2.2. Subcloning for MCF7-IR Cells

We subcloned radiation-surviving MCF-IR cells according to our previous report, where we established radiation-surviving H1299-IR cells [4]. Semi-confluent cultures were exposed to 10 Gy X-rays at room temperature using a linear accelerator ((MHCL-15S, Mitsubishi Electric Co., Tokyo, Japan). Cells were immediately dispersed with trypsin and spread onto Petri dishes (100-mm diameter). After 30 days, all colonies were harvested and established as MCF7-IR cell lines.

### 2.3. Colony Formation Assay

Cells that survived X-ray irradiation were analyzed using a clonogenic assay. The cells in logarithmic phase were irradiated with 6 MV of 10 Gy X-ray, and then they were immediately trypsinized and the viable number of the cells (10,000–100,000 cells) were seeded onto 60-mm dishes. The cells were cultured in DMEM containing 10% FBS and after 14–30 days, the cells were fixed with methanol and stained with 2% Giemsa solution (Kanto Chemical Co. Inc., Tokyo, Japan) to determine the number of colonies per dish. Values were corrected by comparison with the plating efficiency of the untreated cells. The viability was evaluated by dividing the number of colonies after irradiation by the number of colonies of unirradiated cells, which is given in percentage after the correction for plating efficiency.

### 2.4. DNA Microarray Analysis

The parental cells and IR cells were seeded onto 60-mm dishes and cultured for 24 h. Total RNA was extracted from semi-confluent cells using the QIAGEN RNeasy kit (Qiagen, Chatsworth, CA, USA) and was labeled and hybridized onto a human microarray chip that targeted approximately 30,000 human genes. The detected signals were then examined by computer analysis (Agilent Technologies Japan, Ltd., Tokyo, Japan).

### 2.5. Data Analysis

Among the 100 top genes having changed in the expression level more than 1.5-fold up- and down- regulations in microarray data, the information on the chromosomal location of genes was derived from two sources: National Center for Biotechnology Information (NCBI) and Basis Local Alignment Search Tool (BLAST). The relationship between the alteration of gene expression by irradiation and the chromosomal location was graphically indicated.

### 2.6. Irradiated Condition

The logarithmic phase of the cells cultured in the flask on a water-equivalent phantom with a 50-mm-thickness was irradiated with 6 MV therapeutic X-rays (MHCL-15S, Mitsubishi Electric Co., Tokyo, Japan) up to 10 Gy at a dose rate of 250 MU/min at room temperature.

### 2.7. Statistical Analysis

The statistics was analyzed using SPSS version 18.0 (SPSS Inc., Chicago, IL, USA). The comparison between two groups was performed by the Mann–Whitney’s U test. The error bar indicates the standard deviation of the mean (±SD) of triplicate samples. Statistical significance was set at *p* < 0.05. All analyses were performed for duplicate experiments both in H1299 and MCF7.

## 3. Results

### 3.1. Radio-Sensitivity of IR Cells

**Figure 1:**
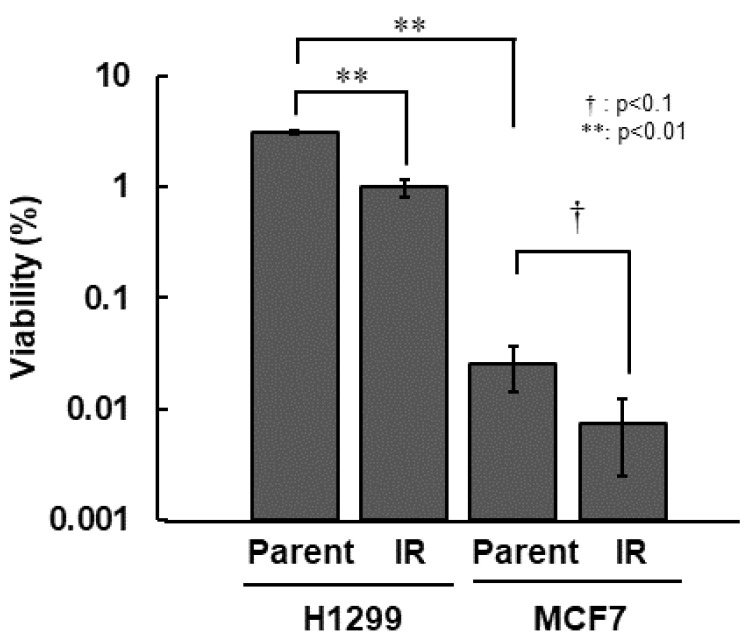
Radio-sensitivity of IR cells. Colony formation assay was performed after 10 Gy irradiation. Cell viability was determined from ratios (%) of viable cells to total numbers of seeded cells. The error bar indicates the standard deviation of the mean of triplicated samples. The double asterisks and the dagger represent statistically significant values of *p* < 0.01 and *p* < 0.1, respectively.

First, we compared all of the radiosensitivity of H1299, H1299-IR, MCF7, and MCF7-IR cells. Among the parental cell lines, MCF7 cells were >100-fold more sensitive than H1299 cells. Both types of IR cells were approximately 3.0-fold more sensitive than their corresponding parental cells (Figure 1 and Table 1).

### 3.2. Chromosomal Location of Upregulated Genes in IR Cells from Microarray Data

**Figure 2:**
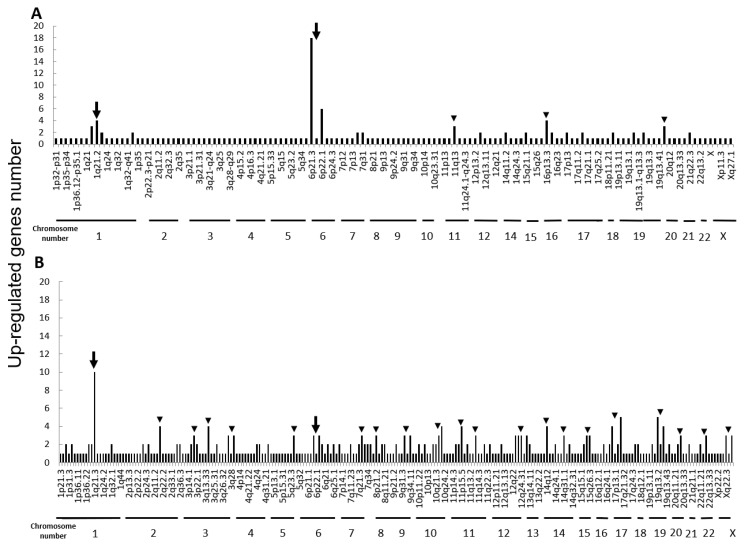
Chromosomal location of up-regulated genes in IR cells from microarray data. Up-regulated gene numbers are displayed on the vertical axis and genome locations from chromosome 1 to chromosome X are plotted along the horizontal axis. (**A**) The human non-small cell lung cancer cell line H1299. (**B**) The human breast cancer cell line MCF7. The arrowheads represent chromosomal locations where three or more differentially expressed genes are present, and the arrow represents the loci that are common to both H1299-IR and MCF7-IR cells.

**Figure 3:**
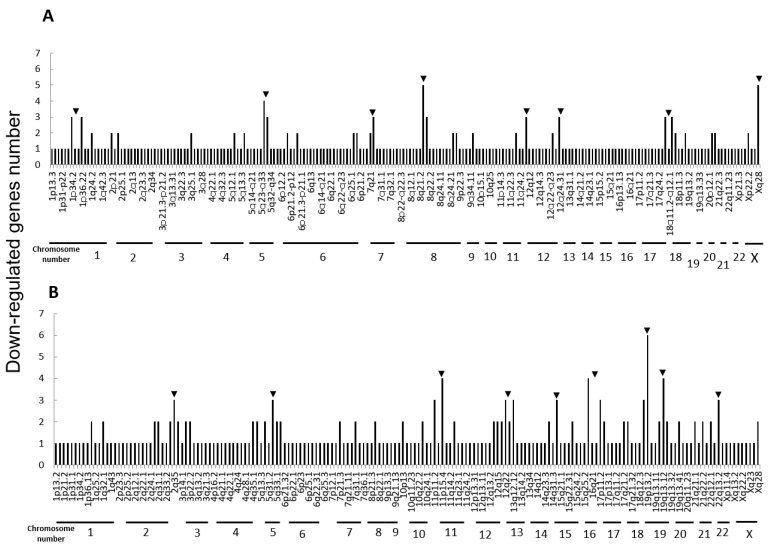
Chromosomal location of down-regulated genes in IR cells from microarray data. (**A**) The human non-small cell lung cancer cell line H1299. (**B**) The human breast cancer cell line MCF7. The arrowheads represent chromosomal locations where three or more differentially expressed genes are present.

Next, we performed microarray analyses (Agilent) on all four cell lines. Comparisons of the change of the gene expression between parental cells and IR cells show 173 up-regulated and 240 down-regulated in H1299-IR cells, and 350 up-regulated and 237 down-regulated genes in MCF7-IR cells. The numbers of differentially expressed genes in IR cells were matched to their corresponding chromosomal loci (Figure 2 and Figure 3). The arrowheads indicate the chromosomal locations where three or more differentially expressed genes are present. Although there were no clustered chromosomal loci in the down-regulated gene, the loci 1q21 and 6p21–22 seemed to be common cluster regions on chromosomes where the gene expression was up-regulated in both H299-IR and MCF7-IR cells.

### 3.3. Functional Categories of Altered Genes

We categorized the functions of the altered genes located in chromosomes 1q21 and 6p21–22 (Table 2 and Table 3). However, there were no common or related genes between H1299-IR and MCF7-IR cells for both up-regulated and down-regulated genes.

## 4. Discussion

In the present study, we found a clustered chromosomal region where gene expression was altered after high-dose X-ray irradiation (Figure 2 and Figure 3). For up-regulated genes, these cluster regions are commonly seen in H1299-IR and MCF7-IR cells on chromosomes 1q21 and 6p21–22 (arrows in Figure 2). In contrast, the clusters in the common loci between H1299 and MCF7 were not shown in the down-regulated genes (Figure 3). Among the common chromosomal sites, 6p21–22 in H1299-IR cells is the location where up-regulated genes are particularly large in number (*n* = 18) (Figure 2). Similarly, the largest number of up-regulated genes in MCF7-IR cells was seen on 1q21 (*n* = 10). Note that 6p21 is a well-known fragile site [13,14]. Such fragile sites have been studied mainly in carcinogenesis associating with tumor malignancies [15,16,17]. For example, 6q21 is commonly deleted in leukemias and primary breast cancer [16,17], and also contains the region for HECT domain and Ankyrin repeat containing E3 ubiquitin-protein ligase 1 (HACE1) gene that under expressed in Wilm’s tumor [18,19]. 6p21 is the largest region of the human genome that encodes about 230 genes in 3.6 megabases (Mb) of the histocompatibility complex (MHC). Therefore, physically, DNA would be easily attacked by irradiation in a large region of 6p21–22 and would cause the alternation of the tumor characteristics especially to the tumor malignancy.

In chromosome 1q21, 12 and 4 up-regulated genes were found in MCF7-IR and H1299-IR cells, respectively (Table 2). The 1q21 region also plays an important role in the development and progression of tumors [20]. For example, in ovarian cancer and neuroblastoma, 1q21–22 is associated with a drug-resistant phenotype and a poorer response to chemotherapy [21,22]. Thus, it is a reasonable hypothesis that genes located at 1q21 might also affect radio-sensitivity.

Chromosomal fragile sites are defined as regions of the genome that exhibit gaps or breaks on metaphase chromosomes under conditions of partial replication stress [23], and it is particularly susceptible to DNA breakage [24]. To date, more than 100 fragile sites have been identified [25]. The common fragile site, FRA6F, is also located at 6q21 [25,26]. While, in the nuclei, loci in each chromosome are especially well ordered according to the chromosome territories (CTs) [27,28]. CT epigenetically affects global gene expression and regulation of the genome, including DNA methylation, remodeling, and other modifications [27,29]. There is one experiment that when cells that pulse are labeled with ^3^H-thymidine, the labeling region of DNA is not labeled in smaller amounts on several chromosomes but is instead restricted to one of the chromosomes [27]. That means ^3^H-thymidine interacts only with the limited region of the DNA. As the interaction between ionizing radiation and DNA occur stochastically depending on conditions such as dose, linear energy transfer, flux density, and oxygen conditions, the frequency of DNA damage depends on linear energy transfer to DNA and the number of secondary electrons and generated oxygen radicals [1,11,12]. These indicate the possibility that limited CTs may be attacked by ionizing radiation, the same as in the ^3^H-thymidine labeling experiment.

According to the functions of the genes located on chromosome 1q21 and 6p21–22 listed in Table 1 and Table 2, many genes encoding histone regulatory proteins were congregated at these loci in H1299 (Table 2 and Table 3), and radiation-induced alteration of gene expression may affect some activities of histone-associated proteins. Also, as some tumor-suppressor associating genes and the genes associating with cellular progression, apoptosis, and the immune system were located in 1q21 and 6p21–22, these chromosomal loci might not only be physically fragile but have the important role for tumor characterization. The possibility that chromosomes 1q21 and 6p21–22 are fragile (stochastically susceptible to attack by ionizing radiation) and the molecular mechanisms of the semi-permanent persistence of the altered gene expression in the IR cells needs to be verified by repeated experiments with the various types of cells in the future.

**Figure 4:**
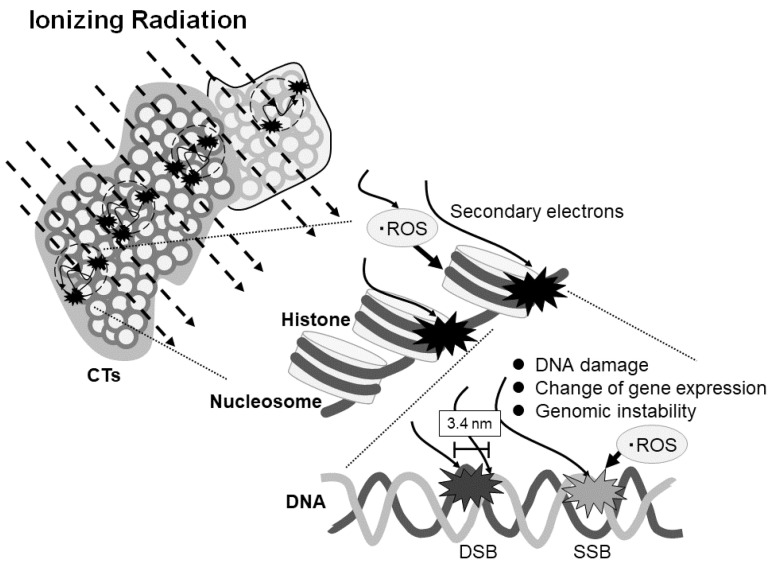
Scheme of the effect of ionizing radiation on DNA damage and gene expression depending on the region of the chromosome territories (CTs). Ionizing radiation stochastically enters the CTs and generates secondary electrons which may cause the reactive oxygen species (ROS). The electrons and ROS can induce single- or double-strand breaks or affect gene expression or genomic stability. DSB and SSB represent double strand break and single strand break, respectively.

In the present report, we have indicated, for the first time, the possibility of ionizing radiation-sensitive DNA fragile sites. These might affect DNA damage, change of temporary or continuous gene expression, and genomic instability (Figure 4). Further study will be necessary to clarify the potential influence of ionizing radiation on gene expression by repeating the experiments with more samples.

## 5. Conclusions

We found a relation between genes with altered expression and their chromosomal loci, especially on 1q21 and 6p21–22, in cells that survived high-dose X-ray irradiation. These chromosome loci might be regarded to be “radio-fragile” sites.

## Figures and Tables

**Table 1:** Radio-sensitivity of Parental cells and IR cells in H1299 and MCF7.

	H1299		MCF7	
	Viability (%)	SEM ^†^	Viability (%)	SEM ^†^
Parent	3.1 × 10^0^	0.050	2.5 × 10^−2^	0.006
IR	9.8 × 10^−1^	0.135	7.5 × 10^−3^	0.003

^†^ SEM represents standard error of the mean.

**Table 2:** Functional categories of up-regulated genes in chromosome 1q21.

Cell Line	Genes and GenBank Accession No.	Functional Category
H1299	histone 2, H2ab (HIST2H2AB) [NM_175065]	Member of the histone H2A family
	S100 calcium binding protein A16 (S100A16) [NM_080388]	*
	hypothetical protein LOC200030 (LOC200030) [NM_183372]	*
	hypothetical protein DJ328E19.C1.1 (DJ328E19.C1.1) [NM_015383]	*
	phosphoprotein enriched in astrocytes 15 (PEA15) [NM_003768]	Insulin resistance in glucose uptake
	histone 2, H2aa (HIST2H2AA) [NM_003516]	Member of the histone H2A family
	hypothetical protein LOC200030 (LOC200030) [NM_183372]	*
	histone 2, H2be (HIST2H2BE) [NM_003528]	Member of the histone H2B family
	histone 2, H2ac (HIST2H2AC) [NM_003517]	Member of the histone H2B family
	small protein effector 1 of Cdc42 (SPEC1) [NM_020239]	*
	hypothetical protein FLJ20519 (FLJ20519) [NM_017860]	*
MCF7	Notch homolog 2 (Drosophila) N-terminal like (NOTCH2NL), [NM_203458]	*
	cDNA FLJ11946 fis, clone HEMBB1000709 [AK022008]	*
	late cornified envelope 2C (LCE2C) [NM_178429]	*
	S100 calcium binding protein A8 (S100A8) [NM_002964]	Cell cycle, progression and differentiation
	BI026064 CM0-MT0374-060201-774-h11 MT0374 [BI026064]	*
	small proline-rich protein 3 (SPRR3), transcript variant 1 [NM_005416]	*
	small proline-rich protein 1A (SPRR1A) [NM_005987]	*
	S100 calcium binding protein A9 (S100A9) [NM_002965]	Inhibition of casein kinase
	cathepsin S (CTSS) [NM_004079]	Degradation of the antigenic proteins to peptides on MHC class II
	S100 calcium binding protein A3 (S100A3) [NM_002960]	*
	S100 calcium binding protein A2 (S100A2) [NM_005978]	Tumor suppressor function
	aquaporin 10 (AQP10) [NM_080429]	Water-selective channel

* Genes whose functional categories are unknown.

**Table 3:** Functional categories of up-regulated genes in chromosome 6p21-22.

Cell Line	Genes and GenBank Accession No.	Functionally Category
H1299	histone 1, H1c (HIST1H1C) [NM_005319]	Member of the histoneH1 family
	histone 1, H1d (HIST1H1D) [NM_005320]	Member of the histoneH1 family
	histone 1, H2bg (HIST1H2BG) [NM_003518]	Member of the histoneH2B family
	histone 1, H2bf (HIST1H2BF) [NM_003522]	Member of the histoneH2B family
	histone 1, H2bc (HIST1H2BC) [NM_003526]	Member of the histoneH2B family
	histone 1, H2be (HIST1H2BE) [NM_003523]	Member of the histoneH2B family
	histone 1, H2ad (HIST1H2AD) [NM_021065]	Member of the histoneH2A family
	histone 1, H2bh (HIST1H2BH) [NM_003524]	Member of the histoneH2B family
	histone 1, H2bd (HIST1H2BD) transcript variant 1, [NM_021063]	Member of the histoneH2B family
	histone 1, H2bb (HIST1H2BB) [NM_021062]	Member of the histoneH2B family
	histone 1, H3b (HIST1H3B) [NM_003537]	Member of the histoneH3 family
	histone 1, H1e (HIST1H1E) [NM_005321]	Member of the histoneH1 family
	immediate early response 3 (IER3), transcript variant short [NM_003897]	Protect cells from apoptosis
	histone 1, H2bi (HIST1H2BI) [NM_003525]	Member of the histoneH2B family
	major histocompatibility complex, class I, C (HLA-C) [NM_002117]	Operate in the immune system
	major histocompatibility complex, class I, B (HLA-B) [NM_005514]	Operate in the immune system
	major histocompatibility complex, class I, F (HLA-F) [NM_018950]	Operate in the immune system
	histone 1, H4c (HIST1H4C) [NM_003542]	Member of the histoneH4 family
	histone 1, H2bk (HIST1H2BK) [NM_080593]	Member of the histoneH2B family
	histone 1, H2ae (HIST1H2AE) [NM_021052]	Member of the histoneH2A family
	histone 1, H2bn (HIST1H2BN) [NM_003520]	Member of the histoneH2B family
	histone 1, H2bl (HIST1H2BL) [NM_003519]	Member of the histoneH2B family
	histone 1, H2bo (HIST1H2BO) [NM_003527]	Member of the histoneH2B family
	histone 1, H2bj (HIST1H2BJ) [NM_021058]	Member of the histoneH2B family
	histone 1, H2bm (HIST1H2BM) [NM_003521]	Member of the histoneH2B family
MCF7	progastricsin (pepsinogen C) (PGC), transcript variant 1 [NM_002630]	*
	ets variant 7 (ETV7) [NM_016135]	Cellular processes on development and differentiation
	proteasome (prosome, macropain) subunit, beta type, 8 [NM_004159]	Processing of class I MHC peptides
	major histocompatibility complex, class II, DP beta 1 (HLA-DPB1), [NM_002121]	Operate in the immune system
	transporter 1, ATP-binding cassette, sub-family B (MDR/TAP) (TAP1), [NM_000593]	Multi-drug resistance
	ubiquitin D (UBD) [NM_006398]	Many cellular processes
	cDNA FLJ37399 fis, clone BRAMY2027587. [AK094718]	Tumor suppressor function
	BE004814 MR2-BN0114-020500-006-e07 BN0114 H [BE004814]	*
	cytidine monophosphate-N-acetylneuraminic acid hydroxylase [NR_002174]	*

* Genes whose functional categories are unknown.

## Data Availability

The data that support the findings of this study are not publicly available. However, data are available from the authors upon reasonable request.
